# A Probabilistic Feature Map-Based Localization System Using a Monocular Camera

**DOI:** 10.3390/s150921636

**Published:** 2015-08-31

**Authors:** Hyungjin Kim, Donghwa Lee, Taekjun Oh, Hyun-Taek Choi, Hyun Myung

**Affiliations:** 1 Urban Robotics Laboratory (URL), Korea Advanced Institute of Science and Technology (KAIST), 291 Daehak-ro (373-1 Guseong-dong), Yuseong-gu, Daejeon 305-701, Korea; E-Mails: hjkim86@kaist.ac.kr (H.K.); leedonghwa@kaist.ac.kr (D.L.); buljaga@kaist.ac.kr (T.O.); 2 Ocean System Engineering Research Division, Korea Research Institute of Ships and Ocean Engineering (KRISO), 32 1312 Beon-gil, Yuseong-daero, Yuseong-gu, Daejeon 305-343, Korea; E-Mail: htchoi@kriso.re.kr

**Keywords:** localization, monocular camera, probabilistic feature map, 3D-to-2D matching correspondences, image data set

## Abstract

Image-based localization is one of the most widely researched localization techniques in the robotics and computer vision communities. As enormous image data sets are provided through the Internet, many studies on estimating a location with a pre-built image-based 3D map have been conducted. Most research groups use numerous image data sets that contain sufficient features. In contrast, this paper focuses on image-based localization in the case of insufficient images and features. A more accurate localization method is proposed based on a probabilistic map using 3D-to-2D matching correspondences between a map and a query image. The probabilistic feature map is generated in advance by probabilistic modeling of the sensor system as well as the uncertainties of camera poses. Using the conventional P*n*P algorithm, an initial camera pose is estimated on the probabilistic feature map. The proposed algorithm is optimized from the initial pose by minimizing Mahalanobis distance errors between features from the query image and the map to improve accuracy. To verify that the localization accuracy is improved, the proposed algorithm is compared with the conventional algorithm in a simulation and realenvironments.

## 1. Introduction

Image-based localization is an important issue in robotics communities as well as computer vision communities. Its applications include navigation of robots or pedestrians, virtual reality, and visualization of tourism cites [1]. Since Google Street view [2] and other tourism sites data sets [3] such as Dubrovnik, Rome, and Vienna are provided, image-based localization has been widely studied and the performance of the suggested approaches has been demonstrated with these data sets. As it is expected to offer enormous image data sets to the entire world on the Internet, image-based localization can be utilized for global localization as a replacement of GPS (global positioning system). As cameras are extensively equipped in everyday electronic devices such as mobile phones, image-based localization is becoming increasingly important in various fields.

In the field of computer vision, Robertson *et al*. [4] proposed an image-based localization method by employing an urban navigation system. They utilized a database of rectified views of building facades by extracting edges of buildings and roads to estimate the pose of a query image. Similarly, Kosecka *et al*. [5] introduced an indoor image-based localization method by matching histograms that are generated by detecting edges of room images. In the robotics field, Nepier *et al*. [6] estimated a robot’s pose by matching road information of a query image to synthetic orthographic images of the road surface produced by a stereo vision system in advance.

color1As robust features have been developed such as SIFT (scale-invariant feature transform) [7] and SURF (speeded up robust features) [8], many researchers are trying to apply the robust features for localization. Zhang *et al*. [9] proposed an image-based localization method in urban environments using triangulation of matched features from database images. 3D feature map-based localization methods are introduced by matching features between a query image and the 3D map which is generated from image databases [10,11]. The accuracy of the feature map is important in feature-based localization methods because errors of the map directly influence the accuracy of localization. In response, the SfM (structure-from-motion) technique [12] was proposed to estimate camera’s motion and scene’s structures from camera images while improving the accuracy of 3D feature maps. color1Bundle Adjustment [13] is one of the widely used optimization methods for minimizing residual errors of the SfM approach. With recent developments of SfM techniques, an SfM model can be constructed on a city-scale considering millions of points [14,15,16]. Accordingly, localization methods based on a 3D feature map such as the SfM model have been widely researched as it has become possible to cover large areas. In large environments, one of the crucial issues is the computation time because the 3D feature map contains millions of features. Lu *et al*. [10] improved computation time by employing kd-tree and nearest neighbor (FLANN; fast library for approximate nearest neighbors) algorithms to find a query feature’s correspondences. Sattler *et al*. [17] applied feature-based localization to a mobile phone platform and suggested a method to accelerate the matching process. Other studies of localization based on a feature map to enhance the accuracy of matching correspondences have also been reported. Irschara *et al*. [18] enhanced registration performance using relevant fragments of a 3D model. Other researchers also improved the performance of 3D-to-2D correct correspondences using mutual visibility information [3] and query expansion [19].

According to [14,17,18], localization methods based on a feature map solve the real-time problem while also providing high performance of matching on a large scale map. However, these studies assume that the map has high accuracy and sufficient features. It therefore remains challenging to apply the feature-based localization methods with insufficient and uncertain features. Since the most feature-based localization methods [3,9,10,11,12,13,14,15,16,17,18,19] use camera pose estimation methods [20,21,22,23,24], uncertainty of features is not considered during the camera pose estimation. color1Although Ferraz *et al*. [25] proposed a camera pose estimation method considering features’ uncertainties by estimating the uncertainties from a bunch of feature points, this method is unsuitable to be employed in a mobile robot application because it is usually not easy to obtain many features of the same object from various views. Thus, a novel localization method is needed to enhance localization accuracy by considering uncertainty of the feature map for mobile robot system during the camera pose estimation.

The uncertain feature map is widely used in SLAM (Simultaneous localization and mapping) techniques in robotics communities. The camera-based SLAM techniques [26,27,28,29] generate a probabilistic feature map based on a sensor system modeling and estimate the robot’s pose at the same time. Since the monocular camera is not able to estimate the full states of the robot’s pose at a single observation, it is required to incorporate additional information such as the robot’s prediction [30] or a scale-known landmark [29,31,32]. There are also researches to estimate the robot’s pose using a pre-built map from the SLAM algorithm, which is called a robot relocation problem. Jeong *et al*. [33] proposed a relocation method of a cleaning robot installed with an upward-looking camera using Harris corners and their orientation information from a ceiling and side walls. This algorithm estimates the robot’s pose using Hough clustering [34] exploiting orientation information of features. Since the uncertainty of the individual feature is not considered, large uncertainties of the features directly influence the accuracy of localization. Lee *et al*. [35] proposed a recursive pose estimation algorithm in kidnapping situation of a cleaning robot using EKF (Extended Kalman Filtering)-based SLAM. The robot’s pose is estimated by recursive EKF updates of the corresponding past robot pose of the best feature-matched frame with a current frame. However, it is hard to estimate an optimized position since this algorithm just matches features based on the single frame. Moreover, these algorithms are designed based on the assumption that the robot is moving on a flat ground, so the robot’s pose can only be estimated in 3-DoF (*x*, *y*, yaw).

Most feature map-based localization methods are performed in a feature map which has sufficient number of features. However, it is challenging to apply these methods in real environment because the feature map from real environment also contains some areas which have inaccurate and insufficient features. The main contribution of this paper is the suggestion of the novel image-based localization method that can cover extensive areas containing inaccurate and insufficient features. In addition, as the proposed method is able to estimate 3D camera pose, it can deal with a relocation situation in a 3D SLAM system. In our approach, a probabilistic feature map is generated from an insufficient data set using sensor modeling. An initial camera pose is estimated by the P*n*P (Perspective-*n*-Point) algorithm [20,22] using 3D-to-2D matching correspondences between the map and the query image. The camera pose is further enhanced by minimizing Mahalanobis distance error between the matching correspondences on its image plane.

The rest of this paper is structured as follows. Section 2 explains the method of generating a probabilistic feature map. In Section 3, the proposed localization method is explained in detail. To verify the effectiveness of the proposed method, the results of simulations and experiments are shown in Section 4. In Section 5, a conclusion and directions for future work are provided.

## 2. Generation of Probabilistic Feature Map

To generate a probabilistic feature map, camera-based sensor data are necessary in advance. If the sensor data and their corresponding poses are provided, a 3D map can be constructed. After features are extracted from each image, it is possible to express a probabilistic feature map in the 3D space by referring to its sensor system modeling. Any camera-based sensor system such as a stereo camera, ToF (time-of-flight) camera, and Kinect can be modeled. In this section, the method to generate a 3D probabilistic feature map is introduced using a camera based on bearing-only landmark initialization [36,37].

### 2.1. Definition of Probabilistic Feature Map

The probabilistic feature map has *m* features that are assumed to have Gaussian distributions comprising position and covariance values. The probabilistic feature map is expressed on the global coordinates of the 3D space as follows:

(1) $$\begin{array}{ccc} \mathrm{Map} & = & \left[F_{1},F_{2},\cdots ,F_{m}\right] \end{array}$$

(2) $$\begin{array}{ccc} F_{i} & = & \left[X_{f_{i}},C_{f_{i}}\right]\;\left(i=1,\cdots ,m\right) \end{array}$$

where $F_{i}$ is the *i*-th feature composed of a position, $X_{f_{i}}\in R^{3}$, and its covariance, $C_{f_{i}}\in R^{3\times 3}$, and *m* is the total number of features. The feature also contains additional information for feature matching such as a descriptor vector and an index of the image. The method of constructing the probabilistic feature map is explained below.

### 2.2. Probabilistic Representation of Features

**Figure 1:**
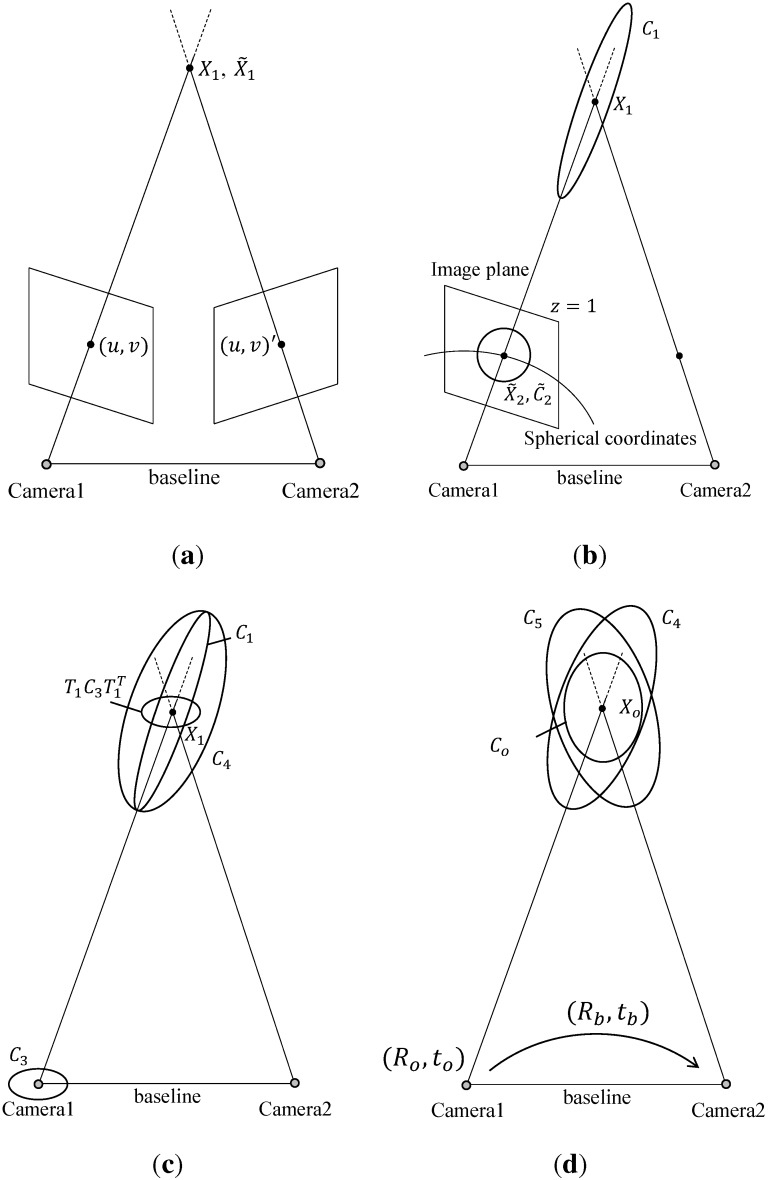
Generation steps of probabilistic features in a stereo system. (**a**) Matched feature points on each image plane; (**b**) Conversion from Cartesian coordinate to spherical coordinate system; (**c**) Estimating covariance from a single camera; (**d**) Estimating initial covariance of the stereo system by merging covariance of each camera.

A probabilistic representation of features is related to a sensor system modeling. Although various image-based sensor systems are available for use, this paper deals with a stereo vision system without loss of generality. Figure 1 shows the steps of probabilistic representation considering the stereo vision system. Let us consider a point on the 3D space represented by $X={\left(x,y,z\right)}^{T}$ in Cartesian coordinates and $\tilde{X}={\left(\rho ,\theta ,\phi \right)}^{T}$ in spherical coordinates. The covariance of each coordinate system is represented by *C* and $\tilde{C}$ as follows:

(3) $$\begin{array}{c} C=\left[\begin{array}{ccc} \sigma_{x}^{2} & \sigma_{xy}^{2} & \sigma_{xz}^{2}\\ \sigma_{xy}^{2} & \sigma_{y}^{2} & \sigma_{yz}^{2}\\ \sigma_{xz}^{2} & \sigma_{yz}^{2} & \sigma_{z}^{2} \end{array}\right],\tilde{C}=\left[\begin{array}{ccc} \sigma_{\rho }^{2} & \sigma_{\rho \theta }^{2} & \sigma_{\rho \phi }^{2}\\ \sigma_{\rho \theta }^{2} & \sigma_{\theta }^{2} & \sigma_{\theta \phi }^{2}\\ \sigma_{\rho \phi }^{2} & \sigma_{\theta \phi }^{2} & \sigma_{\phi }^{2} \end{array}\right] \end{array}$$

where each element denotes the covariance between corresponding axes. By measuring intrinsic parameters and the baseline of two cameras, the position of a feature in the 3D space can be calculated as $X_{1}$ and ${\tilde{X}}_{1}$ on each coordinate system by a triangulation algorithm, as shown in Figure 1a. For estimating the covariance based on the sensor system modeling, a spherical coordinate system is employed, as shown in Figure 1b. As the relationship of covariances between these two coordinate systems is expressed by a Jacobian transformation, the covariance of the spherical coordinate system at $X_{1}$ can be converted to the Cartesian coordinate system as follows (refer to Figure 1b):

(4) $$\begin{array}{c} C_{1}=J_{F}\left(\rho ,\theta ,\phi \right){\tilde{C}}_{1}J_{F}{\left(\rho ,\theta ,\phi \right)}^{T} \end{array}$$

where $J_{F}\left(\rho ,\theta ,\phi \right)$ is the Jacobian matrix at ${\tilde{X}}_{1}$ for converting the spherical coordinate system to the Cartesian coordinate system; *ρ*, *θ*, and *ϕ* denote the radial distance, inclination, and azimuth, respectively. Since the covariance of features on the 3D space is influenced by the depth information, ${\tilde{C}}_{1}$ is formulated as follows:

(5) $$\begin{array}{c} {\tilde{C}}_{1}=\left[\begin{array}{cc} z^{2}\sigma_{1}^{2} & 0\\ 0 & {\bar{C}}_{1} \end{array}\right] \end{array}$$

where $\sigma_{1}$ is the standard deviation of the depth error and ${\bar{C}}_{1}$ is a submatrix of ${\tilde{C}}_{1}$ regarding to *θ* and *ϕ*. As the uncertainty in the radial direction on the spherical coordinate system is dependent on the depth value, the covariance of radial distance is set to $z^{2}\sigma_{1}^{2}$ according to the sensor modeling of a stereo vision system [38]. ${\bar{C}}_{1}$ is influenced by pixel errors of the feature on an image. rcolor1${\tilde{X}}_{2}$ and ${\tilde{C}}_{2}$ are the projected point of $X_{1}$ and its covariance on a virtual image plane where z=1, as shown in Figure 1b. Since the inclination and azimuth of ${\tilde{X}}_{1}$ and ${\tilde{X}}_{2}$ are same, the inclination and azimuth of ${\tilde{C}}_{1}$ are also same as the ones of ${\tilde{C}}_{2}$. Therefore, ${\bar{C}}_{1}$ is equal to the inclination and azimuth covariance of ${\tilde{C}}_{2}$ which is the covariance at ${\tilde{X}}_{2}$. As the uncertainty of features on the image is usually set to a fixed value, the covariance of features on the virtual image plane is expressed as

(6) $$\begin{array}{c} C_{2}=\mathrm{diag}\left(\sigma_{2}^{2},\sigma_{2}^{2},0\right) \end{array}$$

where $\sigma_{2}$ is the standard deviation of the pixel value. The uncertainty in *z*-axis is set to zero because the virtual image plane does not have *z*-axis values. ${\bar{C}}_{1}$ is converted from $C_{2}$ by approximation of the Jacobian matrix on the virtual image plane as follows:

(7) $$\begin{array}{ccc} {\bar{C}}_{1} & = & BJ_{F}\left(x,y,z\right)C_{2}J_{F}{\left(x,y,z\right)}^{T}B^{T} \end{array}$$

(8) $$\begin{array}{ccc} B & = & \left[\begin{array}{ccc} 0 & 1 & 0\\ 0 & 0 & 1 \end{array}\right] \end{array}$$

where $J_{F}\left(x,y,z\right)$ is a Jacobian matrix at $X_{2}$ for converting Cartesian to spherical coordinates on the virtual image plane. To obtain a sub-matrix for the inclination and azimuth, matrix *B* is applied.

Through Equations (3) to (7), the covariance of features from Camera 1, $C_{1}$ in Figure 1b, is estimated. Since the camera pose also contains uncertainty, the uncertainties of the sensor measurement and the camera pose, $C_{1}$ and $C_{3}$ in Figure 1c, are combined by compound approximate transformations [39] as follows:

(9) $$\begin{array}{ccc} C_{4} & = & T_{1}C_{3}T_{1}^{T}+C_{1} \end{array}$$

(10) $$\begin{array}{ccc} T_{1} & = & \left[\begin{array}{cccccc} 1 & 0 & 0 & 0 & z_{1} & -y_{1}\\ 0 & 1 & 0 & -z_{1} & 0 & x_{1}\\ 0 & 0 & 1 & y_{1} & -x_{1} & 0 \end{array}\right] \end{array}$$

where $T_{1}$ denotes approximate transformations to uncertainty of the 3D feature point, $X_{1}$, from the covariance of the camera pose, $C_{3}$. color1$x_{1}$, $y_{1}$, and $z_{1}$ denote the coordinates of the 3D feature point, $X_{1}$. Thus, the covariance from Camera 1 is represented as $C_{4}$ as in Equation (9). Let the rotational components (roll, pitch, and yaw) of the 3D pose be defined as $\theta_{\mathrm{roll}}$, $\theta_{\mathrm{pitch}}$, and $\theta_{\mathrm{yaw}}$. $C_{3}$ can be generally set to $\mathrm{diag}(\sigma_{x}^{2},\sigma_{y}^{2},\sigma_{z}^{2},\sigma_{\theta_{\mathrm{roll}}}^{2},\sigma_{\theta_{\mathrm{pitch}}}^{2},\sigma_{\theta_{\mathrm{yaw}}}^{2})$ from the uncertainty of each element (*x*, *y*, *z*, roll, pitch, and yaw).

By estimating the covariance of features, the features can now be expressed as Gaussian distributions. Since there are two Gaussian distributions of the features from two cameras in the stereo system, a merging method of multivariate Gaussian distributions is required for estimating one Gaussian distribution for a single observation from this system. When two Gaussian distributions are $N(\mu_{1},\Sigma_{1})$ and $N(\mu_{2},\Sigma_{2})$, the merged Gaussian distribution, $N(\mu_{3},\Sigma_{3})$, can be estimated using the merging method [39] as follows:

(11) $$\begin{array}{ccc} \Sigma_{3} & = & {\left(\Sigma_{1}^{-1}+\Sigma_{2}^{-1}\right)}^{-1} \end{array}$$

(12) $$\begin{array}{ccc} \mu_{3} & = & \Sigma_{3}\left(\Sigma_{1}^{-1}\mu_{1}+\Sigma_{2}^{-1}\mu_{2}\right) \end{array}$$

In Figure 1d, $C_{4}$ and $C_{5}$ are covariances of the features from Camera 1 and 2, respectively. $C_{5}$ seen from Camera 1 is represented as $C_{5}^{\prime }=R_{b}C_{5}R_{b}^{T}$ where $R_{b}$ is the rotation matrix from Camera 1 to Camera 2. Letting the probabilistic feature from each camera be $N(\mu_{1}=X_{1},\Sigma_{1}=C_{4})$ and $N(\mu_{2}=X_{1},\Sigma_{2}=R_{b}C_{5}R_{b}^{T})$ in Equations (11) and (12), the probabilistic feature is estimated from a single observation of the stereo vision system, as shown in Figure 1d. Through this process, the position and covariance of each feature from a single observation, denoted by $X_{o}$ and $C_{o}$, respectively, can be obtained.

### 2.3. Constructing a Probabilistic Feature Map

To construct a probabilistic feature map, the features from a single observation require transformation to the global coordinate system and merging of the same features from other observations. color1According to [40], a probabilistic feature from a single observation can be transformed to the global coordinate system as follows:

(13) $$\begin{array}{c} F_{o}=\left[R_{o}X_{o}+t_{o},R_{o}C_{o}R_{o}^{T}\right] \end{array}$$

where $R_{o}$ and $t_{o}$ are rotation and translation of Camera 1 to the global coordinate system.

After all features are converted to the global coordinate system, the probabilistic feature map is constructed using clustering and merging methods of the features. The clustering method has two process steps for recognition of identical features. The first process is the feature descriptor matching. color1The descriptor matching method is dependent on the feature extraction algorithm, and the approximate best-bin-first search method [41] is employed as a matching method for SURF and SIFT descriptors. However, feature matching includes many outliers. Thus, the geometric constraint is employed to remove outliers of matching in the next step. To check the geometric constraint of each feature, the Bhattacharyya distance metric [42] is used by measuring the geometric distance between the probabilistic representations of two features. As the Bhattacharyya distance measures a distance between two probability distributions, the Bhattacharyya distance for two multivariate Gaussian distributions, $N(\mu_{1},\Sigma_{1})$ and $N(\mu_{2},\Sigma_{2})$, is

(14) $$\begin{array}{ccc} D_{B} & = & \frac{1}{8}{\left(\mu_{1}-\mu_{2}\right)}^{T}\Sigma^{-1}\left(\mu_{1}-\mu_{2}\right)+\frac{1}{2}\ln\frac{\left|\Sigma \right|}{\sqrt{|\Sigma_{1}\left|\right|\Sigma_{2}|}} \end{array}$$

(15) $$\begin{array}{ccc} \Sigma & = & \frac{\Sigma_{1}+\Sigma_{2}}{2} \end{array}$$

where |·| denotes determinant. If the Bhattacharyya distance of two features is lower than a certain threshold, those features are regarded as the same features. After finding the same features by checking the descriptor matching and the geometric constraint, probabilistic features are estimated by the merging method described in Equations (11) and (12). Through the merging method of the same features, the probabilistic feature map is constructed.

## 3. Localization Method Using Probabilistic Feature Map

Using prior collected data, the probabilistic feature map can be generated in advance. With this pre-built map, the localization problem is solved using only a monocular camera. The localization of the camera is performed by matching a query image of the camera to the probabilistic feature map.

### 3.1. Generation of Matching Correspondences

It is difficult to find matching correspondences between a query image and the map with robustness, speediness, and accuracy in a large scale area. Many studies have attempted to solve this problem [1,10,17,18,19]. This paper therefore does not deal with matching correspondences deeply and instead applies a simple matching method.

To find matching correspondences between the query image and the probabilistic feature map, features are extracted from the query image. First, candidates of matching correspondences are generated from matched features between the query image and the feature map by descriptor matching. Although descriptor matching of features using SURF or SIFT is still reliable, there are many outliers due to the existence of identical descriptors with different features. Thus, the epipolar constraints-based RANSAC algorithm [43] is utilized to remove mismatched correspondences. As a result, the matching correspondence of features on the query image and the map is expressed as follows:

(16) $$\begin{array}{c} \mathrm{pairs}=\left[q_{j},F_{j}\right]\;\left(j=1,\cdots ,n\right) \end{array}$$

where $q_{j}\in R^{2}$ denotes the *j*-th feature position from a query image and $F_{j}\in R^{3}$ denotes the matched probabilistic feature consisting of its position $X_{f_{j}}$ and covariance $C_{f_{j}}$, and *n* is the total number of the matching correspondences.

### 3.2. Projection of Probabilistic Feature onto Image Plane

**Figure 2:**
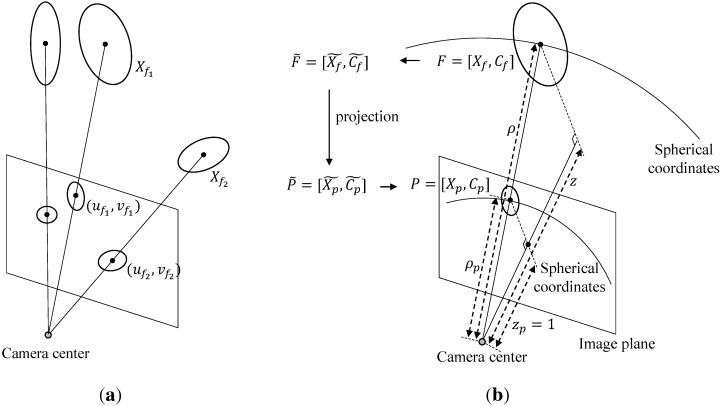
(**a**) The projection results of probabilistic features on the map; (**b**) The projection of the probabilistic feature on the map onto the image plane using spherical coordinates.

The probabilistic features on the map can be projected onto the image plane by utilizing intrinsic and extrinsic parameters of the camera, as shown in Figure 2a. A point in the 3D space can be easily projected to the 2D image plane [43] whereas the covariance in the 3D space cannot. Since the projection onto the image plane is a non-linear transformation, the Jacobian approximation method is employed to project the covariance, in a similar manner to that described in Equations (4) and (7) in Section 2.

The projection process of a probabilistic feature is shown in Figure 2b. When the camera pose is located at the origin, $X_{f}={\left(x,y,z\right)}^{T}$ and ${\tilde{X}}_{f}={\left(\rho ,\theta ,\phi \right)}^{T}$ are easily projected into the virtual image plane as follows:

(17) $$\begin{array}{c} X_{f}={\left(x,y,z\right)}^{T}\to X_{p}={\left(x_{p},y_{p},z_{p}\right)}^{T}={\left(\frac{x}{z},\frac{y}{z},1\right)}^{T} \end{array}$$

(18) $$\begin{array}{c} {\tilde{X}}_{f}={\left(\rho ,\theta ,\phi \right)}^{T}\to {\tilde{X}}_{p}={\left(\rho_{p},\theta_{p},\phi_{p}\right)}^{T}={\left(\frac{\rho }{z},\theta ,\phi \right)}^{T} \end{array}$$

where $X_{p}$ and ${\tilde{X}}_{p}$ are the corresponding points on the virtual image plane in Cartesian and spherical coordinates, respectively; *ρ* denotes the distance between the camera origin and $X_{f}$; and $\rho_{p}$ denotes the distance between the camera origin and $X_{p}$ as shown in Figure 2b. When projected onto the image plane, the inclination and azimuth are not changed in spherical coordinates. Using this property, the covariance in the 3D space can be projected onto the image plane approximately. The projection steps for the covariance of a feature are $C_{f}\to {\tilde{C}}_{f}\to {\tilde{C}}_{p}\to C_{p}$. First, the 3D covariance of a feature on the Cartesian coordinate system is converted into the spherical coordinate system by using a Jacobian matrix as follows:

(19) $$\begin{array}{c} {\tilde{C}}_{f}=J_{F}\left(x,y,z\right)C_{f}J_{F}{\left(x,y,z\right)}^{T} \end{array}$$

where $C_{f}$ and ${\tilde{C}}_{f}$ are covariance matrices on the Cartesian and spherical coordinates; and $J_{F}\left(x,y,z\right)$ is a Jacobian matrix for converting Cartesian to spherical coordinates at $X_{f}$. In the projection step, ${\tilde{C}}_{f}\to {\tilde{C}}_{p}$, in the spherical coordinate system, it can be seen that ${\tilde{C}}_{p}={\tilde{C}}_{f}$ since the inclination and azimuth are not changed in the spherical coordinate system and the radial distance can be neglected due to the fact that depth values of points in the image plane is unity (z=1). Next, the covariance in spherical coordinates is converted to Cartesian coordinates on the virtual image plane as follows:

(20) $$\begin{array}{c} C_{p}=J_{F}\left(\rho ,\theta ,\phi \right){\tilde{C}}_{p}J_{F}{\left(\rho ,\theta ,\phi \right)}^{T} \end{array}$$

where $J_{F}\left(\rho ,\theta ,\phi \right)$ is a Jacobian matrix at ${\tilde{X}}_{p}$. As $X_{p}$ and $C_{p}$ denote the position and the covariance on the virtual image plane in the 3D space, they are converted to image coordinates by utilizing the intrinsic parameter of the camera as follows [43]:

(21) $$\begin{array}{ccc} {\bar{X}}_{p} & = & BKX_{p},\;{\bar{C}}_{p}=BKC_{p}{\left(BK\right)}^{T} \end{array}$$

(22) $$\begin{array}{ccc} B & = & \left[\begin{array}{ccc} 1 & 0 & 0\\ 0 & 1 & 0 \end{array}\right] \end{array}$$

where *K* denotes the intrinsic parameter of a camera. To obtain sub-matrices of $X_{p}$ and $C_{p}$ for *x* and *y*, the matrix *B* is applied.

Through these steps, the points and covariances of features in the map are projected onto the image. Let us define the projection operator P of a point and covariance as follows:

(23) $$\begin{array}{c} {\bar{P}}_{f}=\left({\bar{X}}_{p},{\bar{C}}_{p}\right)=P\left(X_{f},C_{f}\right) \end{array}$$

where $\left(X_{f},C_{f}\right)\in R^{3}\times R^{3\times 3}$ and $\left({\bar{X}}_{p},{\bar{C}}_{p}\right)\in R^{2}\times R^{2\times 2}$ denote probabilistic features on the 3D map and on the image, respectively. Therefore, all probabilistic features on the 3D map can be represented probabilistically on a virtual image plane through the projection operator P without considering the camera’s extrinsic parameter.

### 3.3. Estimating Camera Pose Based on Probabilistic Map

The camera pose of the query image is estimated using 3D-to-2D matching correspondences. After features are extracted from the query image, correspondences between the probabilistic feature map and the query image are acquired by Equation (16). Once color13D-to-2D matching correspondences are offered, the P*n*P algorithm [20,21] which is one of the most widely used algorithms for the camera pose estimation, is employed for comparison with the proposed algorithm. The P*n*P algorithm is also used for the initial point generation of the proposed algorithm. The P*n*P algorithm estimates *R* and *t* of the camera pose by minimizing the error of 3D-to-2D matching correspondences from the relation equation as follows:

(24) $$\begin{array}{c} \left\{R,t\right\}=\arg\min_{R,t}\sum_{j=1}^{n}e_{j}\left(q_{j},KRX_{f_{j}}+t\right) \end{array}$$

where $q_{j}$ and $X_{f_{j}}$ are defined in Equation (16), *K* is the camera’s intrinsic parameter, $e_{j}$ denotes the distance error of each pair, *n* is the total number of the matching correspondences. The proposed algorithm considers the probabilistic information of the map during localization by minimizing Mahalanobis distance errors between the correspondences. Since a point on the image only represents a ray on the 3D space, it is difficult to employ the Mahalanobis distance in the 3D space. Therefore, we propose a method to employ the Mahalanobis distance on a 2D image where the probabilistic features are projected. Since the camera pose is required for performing projection of probabilistic features onto the 2D image, the initial pose is necessary. Thus, the conventional P*n*P algorithm is utilized for the initial pose estimation.

All matched features from the probabilistic feature map, $F_{j}$ from Equation (16) can be projected onto the image plane by Equation (23). However, Equation (23) does not consider the camera’s pose. Considering the camera pose, probabilistic features are projected onto the image plane at certain *R* and *t* as follows:

(25) $$\begin{array}{c} {\bar{P}}_{F_{j}}{{|}_{R,t}=\left({\bar{X}}_{p},{\bar{C}}_{p}\right)|}_{R,t}=P\left(R^{T}X_{f_{j}}-R^{T}t,R^{T}C_{f_{j}}R\right) \end{array}$$

where ${\bar{P}}_{F_{j}}{|}_{R,t}$ indicates the projected probabilistic feature on the image. Using Equations (16) and (25), the Mahalanobis distance, $D_{M}$, on the image plane between the projected probabilistic feature at certain *R* and *t* and the extracted feature from the query image is expressed as:

(26) $$\begin{array}{c} D_{M}\left(q_{j},{\bar{P}}_{F_{j}}{|}_{R,t}\right)=\sqrt{{\left(q_{j}-{\bar{X}}_{p}{|}_{R,t}\right)}^{T}{\left({\bar{C}}_{p}{|}_{R,t}\right)}^{-1}\left(q_{j}-{\bar{X}}_{p}{|}_{R,t}\right)} \end{array}$$

Minimizing the Mahalanobis distance errors for all correspondences, the optimal solution can be obtained as follows:

(27) $$\begin{array}{c} \left\{R,t\right\}=\arg\min_{R,t}\frac{1}{N}\sum_{j=1}^{N}\min\left(D_{M}\left(q_{j},{\bar{P}}_{F_{j}}{|}_{R,t}\right),\tau \right) \end{array}$$

where $F_{j}$ and $q_{j}$ are the matching correspondences from Equation (16); and *τ* denotes a certain threshold to restrict the maximum Mahalanobis distance. color1Although mismatched pairs are rejected using the epipolar constraints-based RANSAC algorithm [43], there might still be mismatches. The localization accuracy is improved by the maximum restricted Mahalanobis distance that reduces influence from outlier matching during optimization. Since Equation (27) is a nonlinear problem with respect to *R* and *t*, various nonlinear optimization algorithms can be employed. Since it is important to set the initial point in the nonlinear optimization, the estimation from the conventional P*n*P method is utilized.

## 4. Simulation and Experiments

The proposed algorithm is validated through comparison with the conventional algorithm in simulations and real experiments. The proposed algorithm is demonstrated in a virtual probabilistic map in the simulation environment. Through testing in the real environment, the robustness of the proposed algorithm is also verified.

### 4.1. Simulation

**Figure 3:**
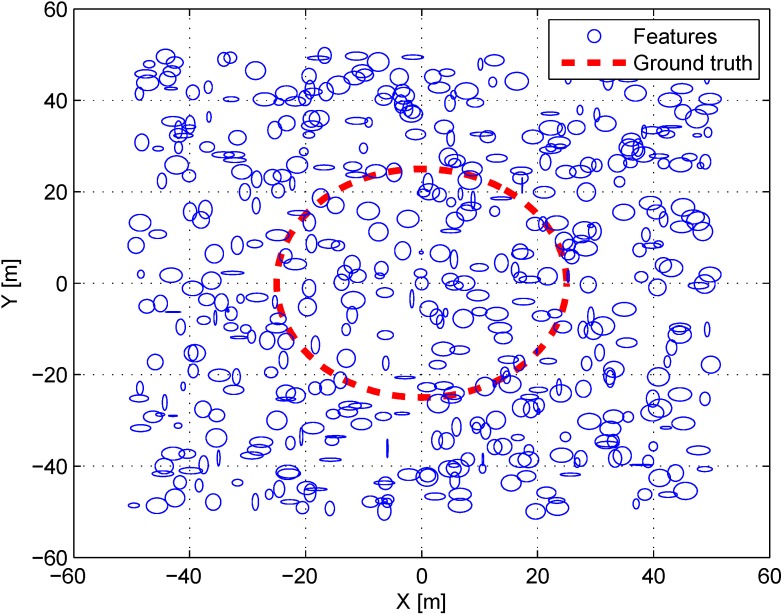
Top view of the simulation environment. Blue circles denote the covariance of features and the red dots denote the ground truth of the robot’s trajectory.

The camera pose is estimated using the proposed algorithm based on the probabilistic feature map. It is assumed that there are feature maps expressed by Gaussian distributions in a virtual 3D space. The features are randomly generated in the virtual 3D space sized 50 m × 50 m × 10 m, similar to a real test environment. Their covariances are set randomly from 1 m to 3 m for each axis. The circular path for a 6-DoF (Degree-of-freedom) camera pose is generated with diameter of 25 m. Figure 3 shows the top view of the virtual environment. The blue ellipses denote the randomly generated covariances of features. The red line is the trajectory of the camera pose. Considering the intrinsic camera parameter and image size, the feature map is projected to the image plane at the virtual path. The feature map contains Gaussian noises based on the covariance of each feature. The features on the image plane have fixed Gaussian noises considering pixel values. 10% of mismatching is also implemented to the matching correspondence between the map and the image plane, similar to real environments.

**Figure 4:**
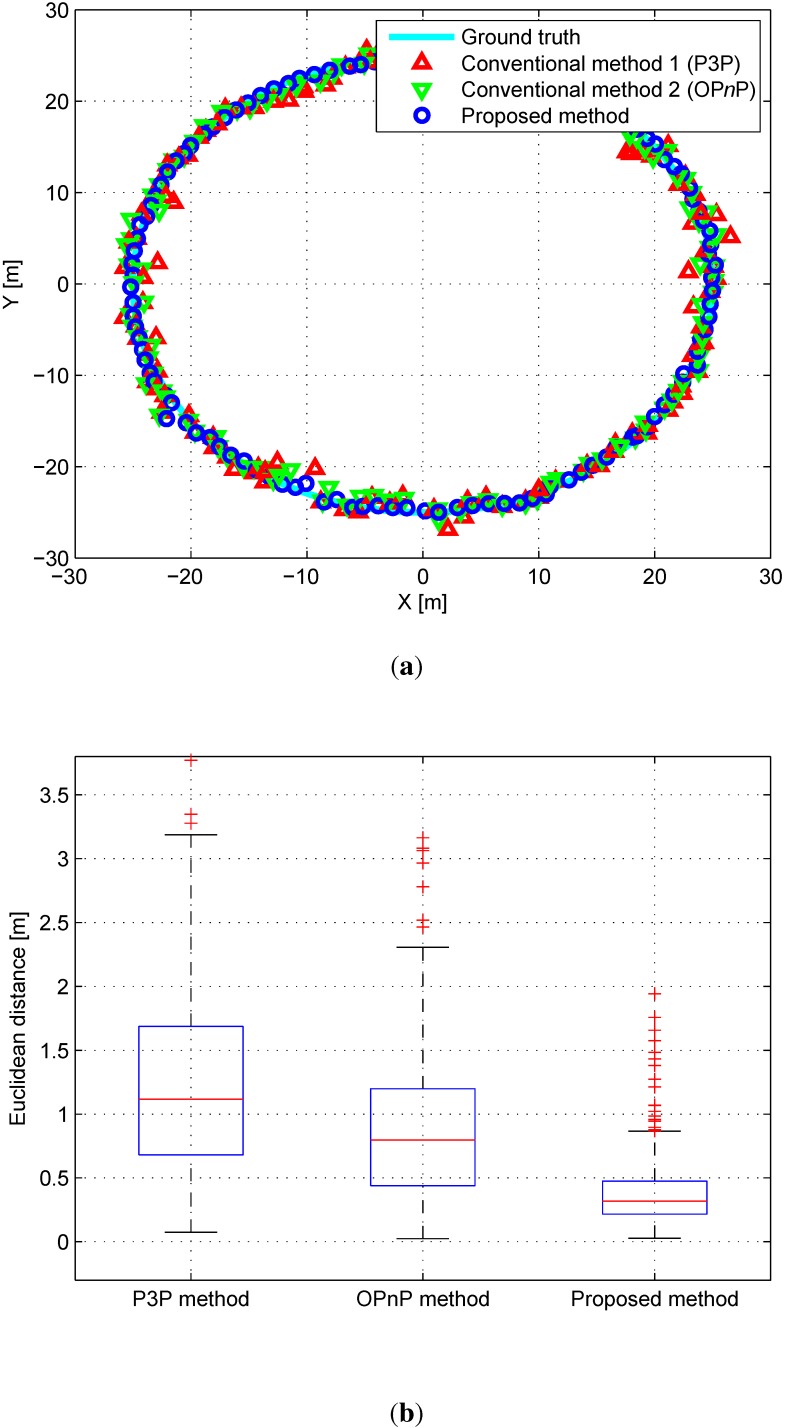
(**a**) Top view of the simulation results. Red triangles, green triangles, and blue circles denote results of P*n*P, OP*n*P, and the proposed algorithm, respectively; (**b**) Euclidean distance errors relative to the ground truth data.

As conventional P*n*P methods, P3P [20] and OP*n*P [22] methods are employed as a traditional and state-of art methods using these matching correspondences between features on the map and the image plane, respectively. The proposed algorithm minimizes errors between the probabilistic map features and extracted features on the image plane. The active-set algorithm [44] is employed as a nonlinear optimization method in the proposed algorithm. Since it is important to set an initial point in the nonlinear optimization method, the result of the conventional P*n*P method is set as the initial point for optimization. Figure 4a shows the top view of the simulation results. The Euclidean distance errors for the P*n*P and the proposed algorithms are shown in Figure 4b. The mean and standard deviation of the error for each 6-DoF are shown in Table 1.

**Table 1:** Comparison of the error statistics of the conventional and the proposed algorithms in the simulation environment (Unit: m, deg).

	P3P Algorithm		OP*n*P Algorithm		Proposed Algorithm	
	Mean	Stdev	Mean	Stdev	Mean	Stdev
*x*	0.634	0.353	0.486	0.132	0.292	0.085
*y*	0.714	0.351	0.665	0.307	0.279	0.076
*z*	0.801	0.444	0.663	0.245	0.706	0.368
$\theta_{\mathrm{roll}}$	1.368	1.795	0.981	0.705	1.227	1.569
$\theta_{\mathrm{pitch}}$	1.527	2.141	1.292	1.493	1.273	0.658
$\theta_{\mathrm{yaw}}$	1.070	0.934	1.188	0.774	0.493	0.257

As can be seen in Table 1, the accuracy of the proposed algorithm seems to be higher than that of the conventional P*n*P algorithm. *x*, *y*, and $\theta_{\mathrm{yaw}}$ in the proposed algorithm especially have better performance because the simulation is made for a mobile robot moving on a flat ground. To be clear, a paired *t*-Test is performed to analyze the performance statistically. As the *p*-value is 1.1527×$10^{-57}$, the superiority of the proposed algorithm is assured.

### 4.2. Experiment in Indoor Environment

**Figure 5:**
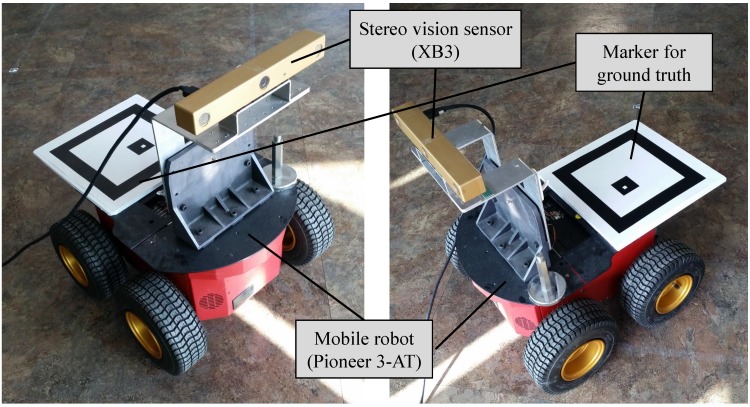
A mobile robot system equipped with a stereo camera. A patterned marker is used for providing ground truth.

**Figure 6:**
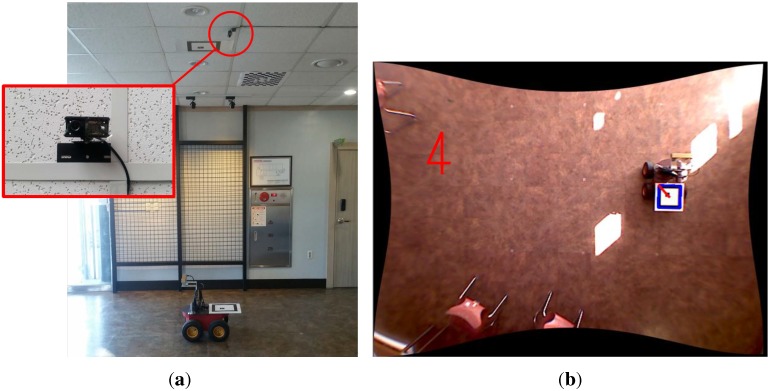
Ground truth system based on global vision sensor. (**a**) Installed camera on the ceiling for the ground truth system; (**b**) The image processing result for ground truth system.

**Figure 7:**
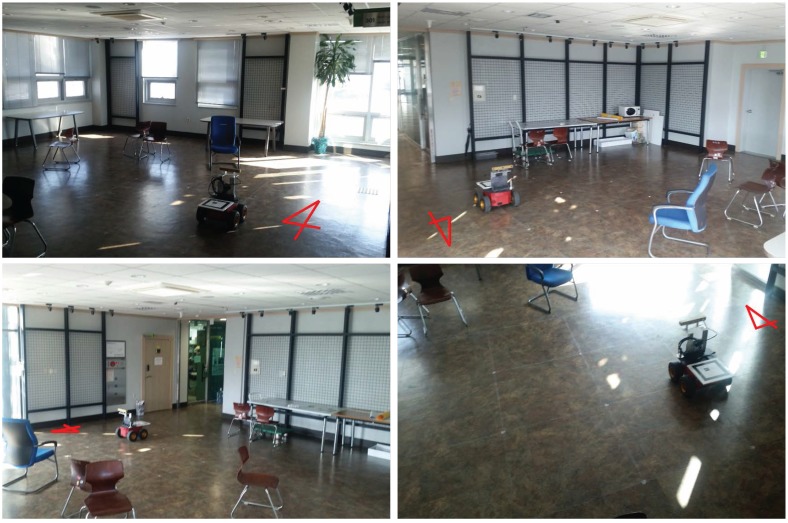
Indoor experiment site for demonstrating the performance of the proposed algorithm.

To validate the performance of the proposed algorithm, experiments with a mobile robot were performed. The mobile robot system, Pioneer 3-AT model [45], is equipped with a stereo vision, Bumblebee XB3 model [46], and a marker system for the ground truth as shown in Figure 5. The XB3 provides the depth value of each pixel. Thus, the sensor produces a 2D image as well as per-pixel depth data with 1920 × 1080 resolution. A camera is installed on the ceiling for measuring the ground truth pose of the mobile robot as shown in Figure 6a and its sample captured image is shown in Figure 6b. The ground truth system is only able to estimate 3-DoF pose (*x*, *y*, $\theta_{\mathrm{yaw}}$). Thus, other elements of robot pose (*z*, $\theta_{\mathrm{roll}}$, $\theta_{\mathrm{pitch}}$) for ground truth are set to zero because the mobile robot is assumed to move on the flat ground. As the resolution of ceiling camera is 640 × 480 pixels and the camera covers 4.4 m × 3.3 m area, the resolution of the ground truth system is about 0.7 cm per pixel. The indoor experiment was performed in the hall as shown in Figure 7. The trajectory of the mobile robot was composed of a circle with a diameter of 2.0 m repeated 5 times. The probabilistic feature map is generated from the first and second laps of the experiment and the proposed localization is demonstrated from third to fifth laps using the pre-generated probabilistic feature map.

**Figure 8:**
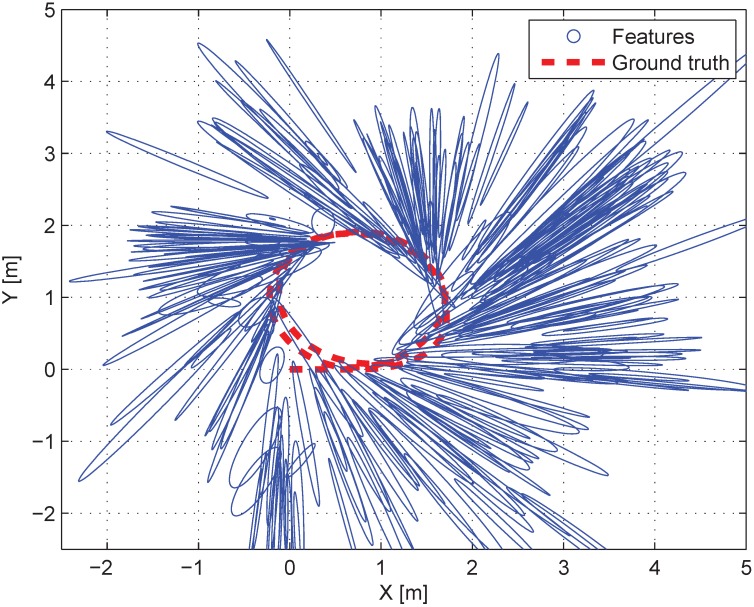
The top view of the indoor environment. Blue circles denote the covariance of features and the red dots denote the ground truth of the robot’s trajectory.

**Figure 9:**
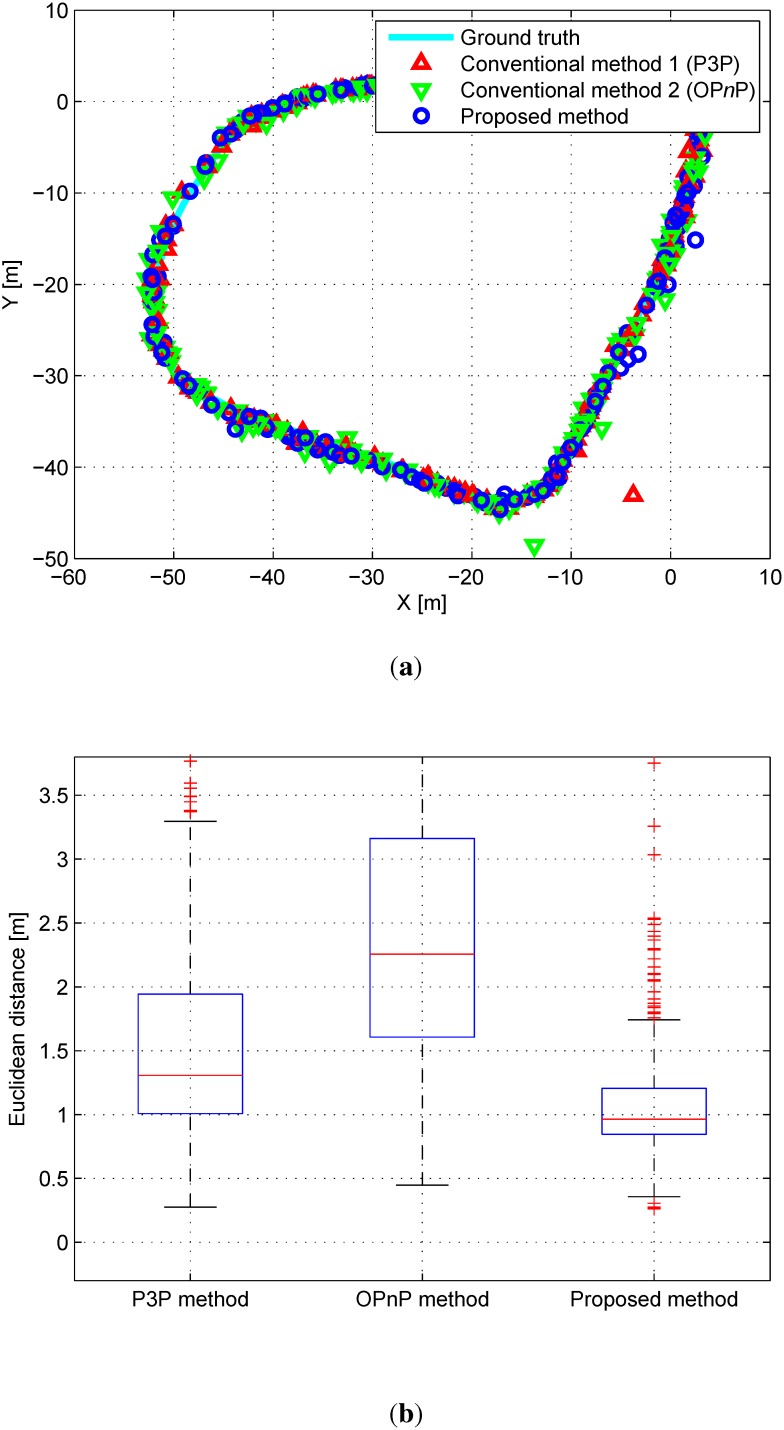
(**a**) Top view of the results of the indoor experiment. Red triangles, color1green triangles, and blue circles denote results of P*n*P, color1OP*n*P, and the proposed algorithm, respectively; (**b**) Euclidean distance errors relative to the ground truth data.

Figure 8 shows the experimental environment where the probabilistic feature map was generated in advance. The shapes of covariance ellipses are mostly narrow since the features were observed only a few times. Figure 9a shows the top view of the results in the indoor experiment. Euclidean distance errors from the ground truth for each algorithm are shown in Figure 9b. The error results of the 6-DoF robot’s pose are presented in Table 2. Similar to the simulation results, the accuracy of the proposed algorithm is higher than that of the conventional algorithm, particularly for the *x*, *y*, and $\theta_{yaw}$ values. As the *p*-value of the paired *t*-Test is 1.5353×$10^{-34}$, the superiority of the proposed method is confirmed statistically.

**Table 2:** Comparison of the error statistics of the conventional and the proposed algorithms in the indoor environment (Unit: m, deg).

	P3P Algorithm		OP*n*P Algorithm		Proposed Algorithm	
	Mean	Stdev	Mean	Stdev	Mean	Stdev
*x*	0.2812	0.0218	0.2752	0.0373	0.224	0.0152
*y*	0.2543	0.0229	0.2813	0.0362	0.2079	0.0131
*z*	0.022	0.0003	0.0641	0.0026	0.0244	0.0003
$\theta_{\mathrm{roll}}$	0.5857	0.2521	1.4794	0.3213	0.7875	0.2246
$\theta_{\mathrm{pitch}}$	0.6502	0.2717	1.3515	0.2914	0.6794	0.2816
$\theta_{\mathrm{yaw}}$	4.9775	5.0588	5.7647	4.5845	2.0417	2.8283

### 4.3. Experiment in Outdoor Environment

**Figure 10:**
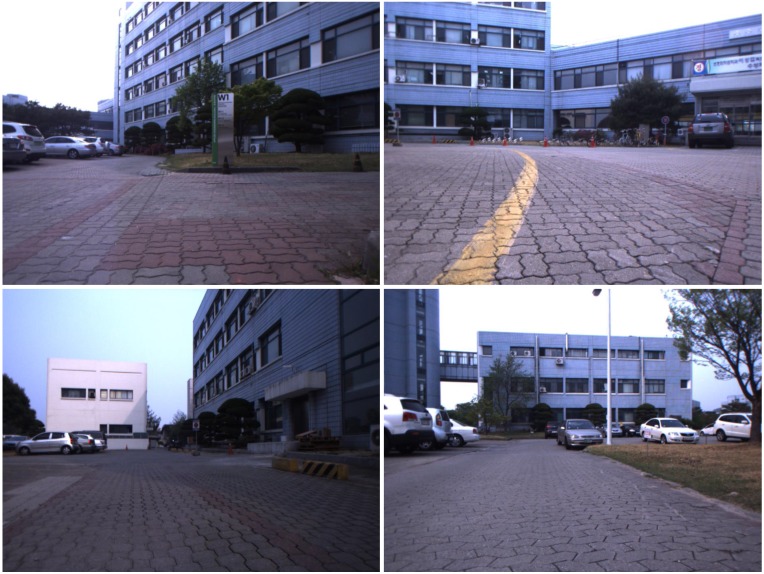
Outdoor experiment site for demonstrating the performance of the proposed algorithm.

**Figure 11:**
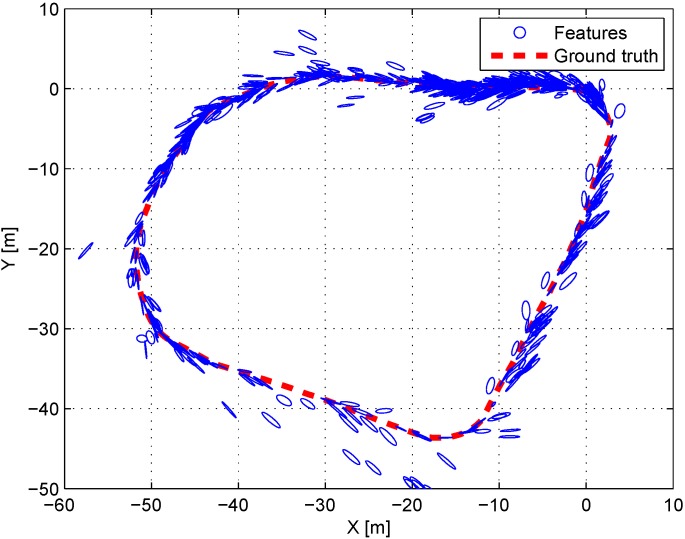
Top view of the outdoor environment. Blue circles denote the covariance of features and the red dots denote the ground truth of the robot’s trajectory.

The outdoor experiments have been conducted at Korea Advanced Institute of Science and Technology in Daejeon, South Korea as shown in Figure 10. color1The robot system for experiments is equipped with the stereo vision same as the previous indoor experiments and we added Huace X90 RTK-GPS receiver [47] and E2BOX IMU 9DOFV2 [48] for the reference which have 2 cm and 1${}^{\circ }$ accuracy, respectively. The probabilistic feature map was collected from 383 positions in 177 m trajectory as shown in Figure 11. The maximum valid depth information from the stereo vision system was restricted to 20 m because the uncertainty of depth data rapidly increases with the distance beyond 20 m.

**Figure 12:**
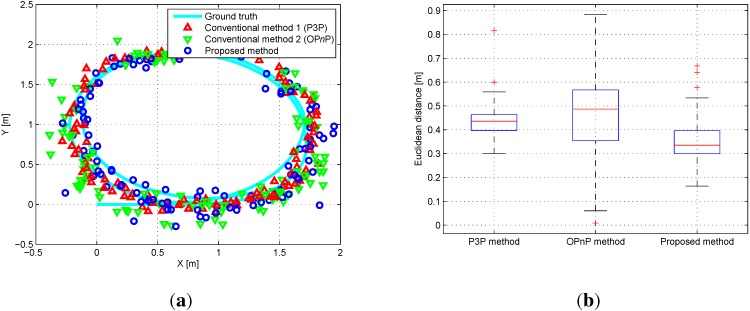
(**a**) Top view of the outdoor results. Red triangles, color1green triangles, and blue circles denote results of P*n*P, color1OP*n*P, and the proposed algorithm, respectively; (**b**) Euclidean distance errors relative to the reference data.

The experiment for localization was performed at 383 positions around the probabilistic feature map. Figure 12a shows the top view of the results in the outdoor experiment. Euclidean distance errors from the reference for each algorithm are shown in Figure 12b. The error results of the 6-DoF robot’s pose are presented in Table 3. The accuracy of the proposed algorithm is also higher than that of the conventional algorithm particularly for the *x*, *y*, and $\theta_{yaw}$ values similar to the simulation and the indoor experiment. It is natural that the accuracy of *z* and $\theta_{\mathrm{roll}}$ values does not show improvement since the *z* and $\theta_{\mathrm{roll}}$ values are not compensated well because the experiment is performed on a flat ground. The experimental results are similar to the simulation results. The *p*-value of the paired *t*-Test is $2.339\times 10^{-23}$, which statistically confirms the superiority of the proposed method.

**Table 3:** Comparison of the error statistics of the conventional and the proposed algorithms in the outdoor environment (Unit: m, deg).

	P3P Algorithm		OP*n*P Algorithm		Proposed Algorithm	
	Mean	Stdev	Mean	Stdev	Mean	Stdev
*x*	1.0865	0.5374	2.1011	1.2546	0.7473	0.1549
*y*	0.8908	0.4334	1.5947	2.0542	0.6935	0.2382
*z*	0.1607	0.0376	0.2056	0.0541	0.1754	0.0356
$\theta_{\mathrm{roll}}$	1.409	1.9777	0.4489	1.1541	2.1761	3.9504
$\theta_{\mathrm{pitch}}$	1.4347	2.4532	1.2055	2.5132	1.3489	2.1354
$\theta_{\mathrm{yaw}}$	2.155	4.264	3.1218	5.1235	1.5689	1.9399

## 5. Conclusions

This paper sought to enhance the accuracy of monocular camera localization using a probabilistic feature map that is generated in advance with a prior data set by adopting probabilistic sensor system modeling. When the map is generated, the probabilistic feature map is estimated not only by the sensor system modeling but also by considering the uncertainty of the robot’s pose. In the conventional P*n*P method, the camera pose is estimated by minimizing the Euclidean distance between 2D-to-3D matching correspondences. The proposed algorithm is optimized based on the Mahalanobis distance error in the image plane between the matching correspondences. The main contribution of this paper is that the proposed method enhances the accuracy of the conventional camera pose estimation algorithm by providing probabilistic sensor modeling. The performance of the proposed algorithm is demonstrated by comparing with the conventional P*n*P algorithm in simulations and real experiments. By the experiments conducted in indoor and outdoor environments, the superiority of the proposed algorithm is proved.

Although the average computation of the P*n*P algorithm takes less than 5 ms per one frame, the proposed algorithm takes about 100 ms per one frame in the computing platform of Intel i7 3.4 GHz with 8 GB RAM. The reason is that the proposed algorithm solves a complex nonlinear optimization problem. color1The average computation times of subtasks are: 187 ms for feature extraction, 231 ms for matching, 5 ms for initial pose estimation using conventional P*n*P algorithm, and 95 ms for optimization in our proposed method. Therefore, a simple linearization method for the proposed algorithm and fast feature management should be researched for real time operation in the future. The proposed algorithm will be also applied to vast data sets and various environments in the future.
